# Bidirectional ventricular tachycardia following ricin intoxication: a case report

**DOI:** 10.1093/ehjcr/ytag115

**Published:** 2026-02-13

**Authors:** Anna Steinacher, Alexandra Schratter, Georg Delle Karth, Philip Eisenburger

**Affiliations:** Department of Internal Medicine and Cardiology, Clinic Floridsdorf, Bruenner Strasse 68, Vienna 1210, Austria; Department of Internal Medicine and Cardiology, Clinic Floridsdorf, Bruenner Strasse 68, Vienna 1210, Austria; Department of Internal Medicine and Cardiology, Clinic Floridsdorf, Bruenner Strasse 68, Vienna 1210, Austria; Department of Internal Medicine and Emergency Medicine, Clinic Floridsdorf, Bruenner Strasse 68, Vienna 1210, Austria

**Keywords:** Ricin intoxication, Bidirectional ventricular tachycardia, Arrhythmia following intoxication, Alternating QRS axis, Case report

## Abstract

**Background:**

Ricin is a highly potent toxin derived from the seeds of the castor oil plant (*Ricinus communis*) and can be lethal even in small amounts. While ricin intoxication is known for its gastrointestinal and systemic toxicity, its potential to induce life-threatening cardiac arrhythmias, such as bidirectional ventricular tachycardia, remains largely unexplored.

**Case summary:**

A previously healthy man ingested 15 castor beans in a suicide attempt and presented to the emergency department with a wide-complex tachycardia at 180 bpm and alternating QRS axis, consistent with bidirectional ventricular tachycardia. Due to severe agitation and suspected aspiration, the patient was endotracheally intubated. As there is no specific antidote for ricin, activated charcoal and intravenous sodium bicarbonate were administered. Attempts at rhythm stabilization, including five electrical cardioversion attempts and a cumulative dose of 450 mg amiodarone, were unsuccessful. After approximately 2 h of supportive care, conversion to sinus rhythm occurred, although short episodes of non-sustained ventricular tachycardia and atrial fibrillation persisted. The patient remained intubated for 10 days due to aspiration pneumonia but subsequently recovered fully. The patient was discharged in good health 24 days after admission.

**Conclusion:**

To the best of our knowledge, this is the first reported case of bidirectional ventricular tachycardia following ricin intoxication. A likely mechanism involves toxin-induced myocardial injury resulting in increased automaticity or triggered activity. Re-entry appears unlikely due to the absence of structural heart disease and the failure of electrical cardioversion. This case underscores the importance of recognizing rare toxin-induced arrhythmias in the differential diagnosis of wide-complex tachycardias, highlighting a need for heightened awareness among cardiologists in the context of acute poisoning.

Learning points

- Ricin intoxication can cause life-threatening ventricular arrhythmias, including the rare occurrence of bidirectional ventricular tachycardia.
- The arrhythmogenic mechanism in ricin poisoning may involve calcium dysregulation and triggered activity, similar to digitalis intoxication or catecholaminergic polymorphic ventricular tachycardia.
- Supportive care and close rhythm monitoring are essential, as guideline-recommended antiarrhythmic measures may fail, yet full recovery is possible.

## Introduction

Ricin, a potent ribosome-inactivating glycoprotein derived from *Ricinus communis* (castor bean), is one of the most toxic naturally occurring substances. Symptoms of ricin intoxication typically include gastrointestinal distress, hypotension, multi-organ dysfunction, and, in fatal cases, multi-organ failure. Various arrhythmias have been reported in the context of ricin poisoning; however, bidirectional ventricular tachycardia (VT) has not previously been described. We report a case of polymorphic/bidirectional VT following ricin intoxication, with discussion of potential underlying mechanisms.

## Summary figure

**Figure:**
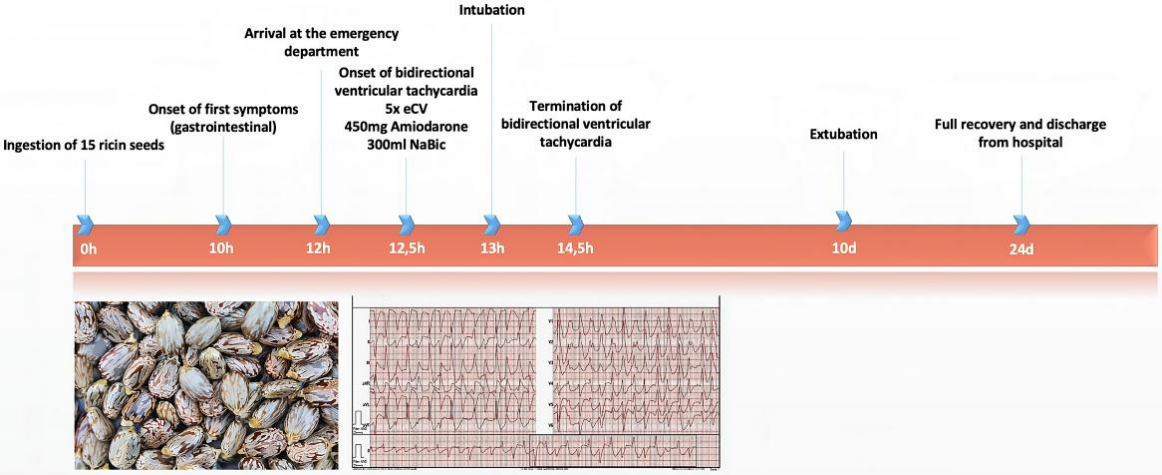


## Case presentation

We report a case of ricin intoxication in a 46-year-old male patient that resulted in bidirectional VT.

A 46-year-old, previously healthy male with a history of depression presented to the emergency department via ambulance with symptoms of severe nausea, vomiting, and diarrhoea after ingestion of ricin seeds in a suicidal attempt. He claimed to have swallowed 15 seeds of the castor oil plant (*Ricinus communis*), which contain high levels of ricin, one of the most potent poisons known.

The patient initially presented agitated, hypotensive, with profuse vomiting as well as diarrhoea and diffuse abdominal pain. Symptoms first appeared approximately 10 h after ingestion. While the heart rate was normal during transport, monitoring in the emergency department showed tachycardia with a heart rate of approximately 180 bpm.

**Figure 1:**
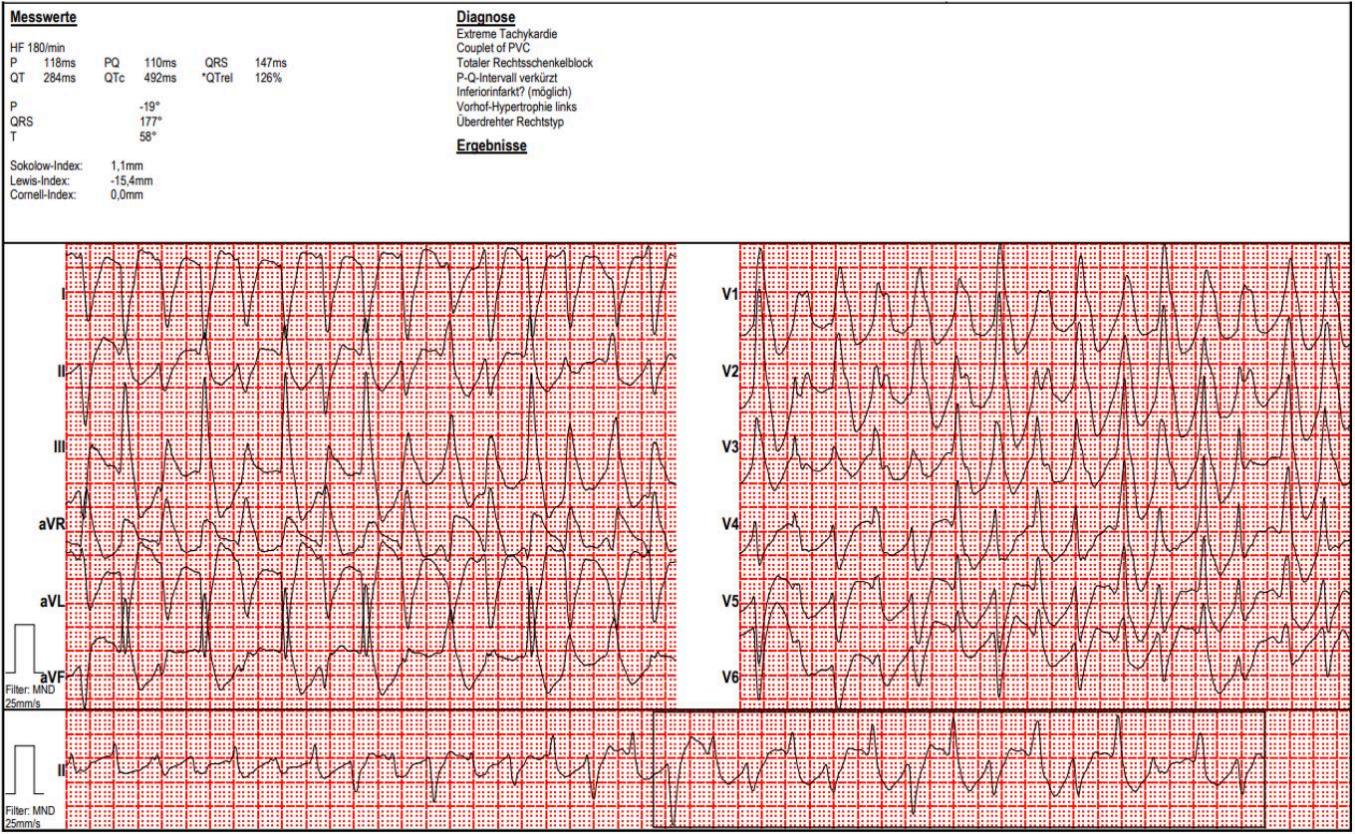
Polymorphic ventricular tachycardia at admission.

A 12-lead electrocardiogram (ECG) revealed rhythmic wide complex tachycardia with beat-to-beat alternation of the QRS axis (*Figure 1*). There was no personal or family history of heart disease or sudden cardiac death.

*Figure 1* shows polymorphic (in serial ECGs, mainly bidirectional) VT (heart rate 180 bpm). There are two alternating QRS-complexes with different axis, both showing right bundle branch block morphology, suggesting two different exits within the left ventricle. Atrioventricular dissociation is present; dissociated P waves can be discerned in lead I.

The first blood gas analysis showed partially compensated metabolic acidosis due to lactic acidosis (pH 7.33, pCO_2_ 18 mmHg = 2.4 kPa, pO_2_ 98 mmHg = 13.1 kPa, base excess −13 mmol/L, HCO_3_^−^ 10 mmol/L, lactate 9.1 mmol/L).

Initial electrolyte values revealed sodium 130 mmol/L, potassium 3.85 mmol/L, and ionized calcium 1.25 mmol/L.

Given that vomiting was refractory to treatment, agitation deteriorated despite sedation, and the patient likely aspirated, rapid sequence induction and intubation were performed to protect the airway. Since there is no specific antidote to ricin, a nasogastric tube was inserted, and activated charcoal was administered.

Efforts to convert the haemodynamically unstable VT, which included a total of five attempts of electrical cardioversion as well as cumulative intravenous administration of 450 mg amiodarone, were unsuccessful, despite adherence to current guideline-recommended management of unstable VT.^1^

Sodium bicarbonate (8.4%) was given in three doses of 100 mL each.

The team was prepared for extracorporeal membrane oxygenation (ECMO) support.

The patient, however, converted to sinus rhythm after 2 h of additional supportive care but continued to have short arrhythmias, such as non-sustained runs of VT and atrial fibrillation.

There were no further cardiac arrhythmias after 72 h after ingestion.

The patient remained intubated and mechanically ventilated for 10 days due to respiratory insufficiency caused by aspiration pneumonia and was subsequently weaned and extubated without complication.

Except for a mild increase in liver enzymes, which resolved 4 days after admission to the intensive care unit, no other organ damage was observed.

Serial troponin measurements showed only mild elevation, which declined rapidly after termination of the arrhythmia. Furthermore, a focused transthoracic echocardiography after termination of the arrhythmia revealed normal left ventricular size and wall thickness, normal left ventricular systolic function, no diastolic dysfunction, and no significant valvular abnormalities. There was no pericardial effusion, and right ventricular function was normal.

**Figure 2:**
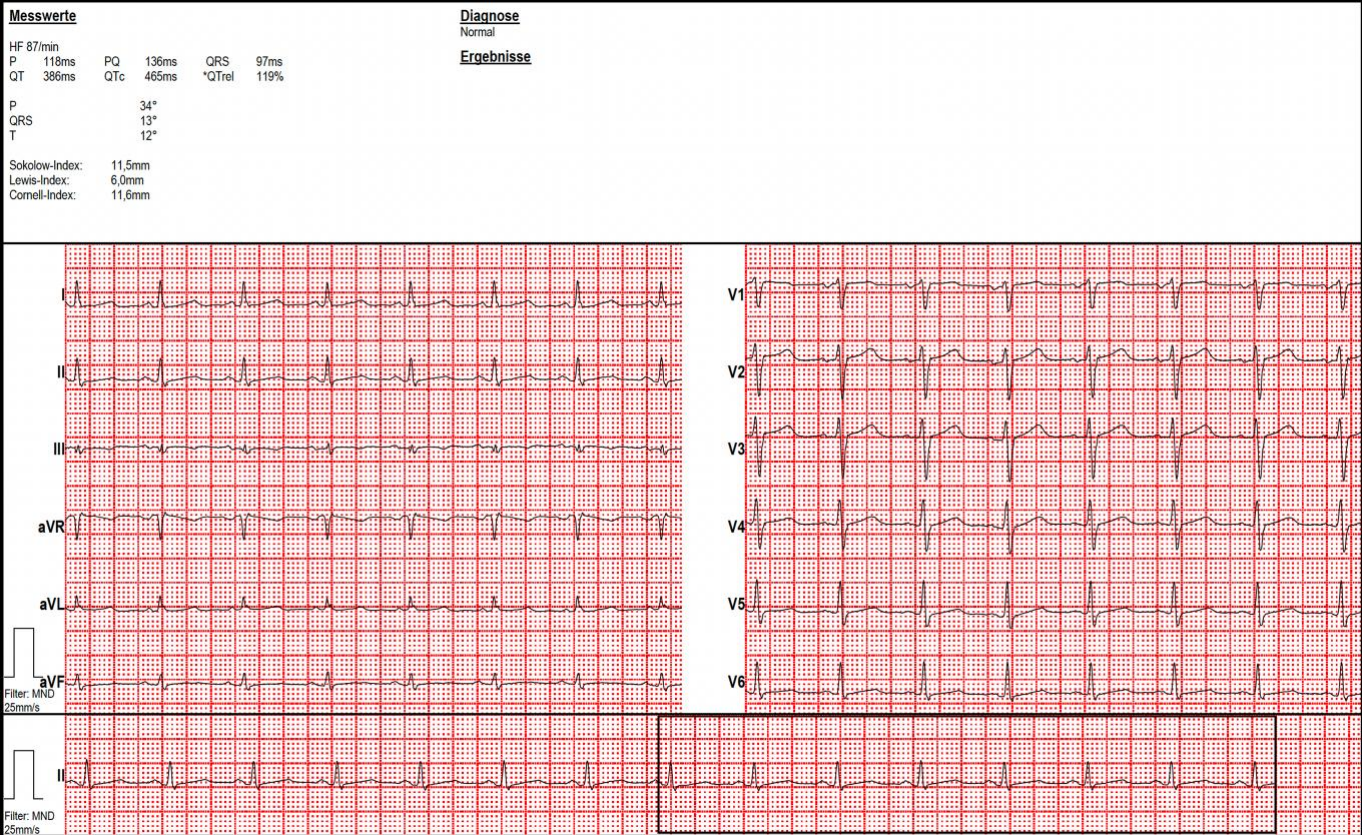
Baseline ECG after recovery.

Baseline ECG did not show any abnormalities (*Figure 2*).

The patient denied intake of any other substances.

Digoxin or digitoxin intoxication, which has been described in the literature as possibly being associated with bidirectional VT,^2^ as well as co-ingestion with other poisons (e.g. aconitine), could be ruled out; digoxin and digitoxin levels were negative at admission.

At discharge, 24 days after admission the patient demonstrated normal left ventricular systolic function on echocardiography, no recurrent arrhythmias, complete resolution of respiratory symptoms after extubation, and no neurological deficits. At follow-up, he reported normal functional capacity in all daily activities.

## Discussion

Ricin is a water-soluble glycoprotein that inactivates ribosomes and inhibits protein synthesis. The median oral lethal dose is 20 mg/kg;^3^ one seed weighs about 500 mg. Our patient had a body weight of 120 kg. If the seeds are swallowed without chewing, the toxic effect can be reduced due to the seeds’ solid coating.^3^

Most of the symptoms occurring after ingestion can be explained by ricin-induced endothelial cell damage, which leads to the so-called vascular leak syndrome, including fluid and protein leakage as well as tissue oedema, ultimately leading to apoptosis.^4^

Symptoms of ricin intoxication typically begin 4 to 6 h after ingestion, and patients may present with abdominal pain, vomiting, diarrhoea, hypotension, hepatic and renal dysfunction, and various types of gastrointestinal bleeding, leading to severe fluid and electrolyte imbalance.^3,5,6^

Various arrhythmias, such as bradycardia, sinus arrhythmia, and tachycardia, have been described in the literature.^6^ In fatal cases, death usually occurs due to multi-organ failure.

To the best of our knowledge, this is the first case of polymorphic/bidirectional VT reported after ricin poisoning.

Regarding the possible mechanisms of bidirectional VT, as described by Almarzuqi *et al*.^7^ we consider the underlying mechanism in our case most likely to be either enhanced automaticity or triggered activity, or a combination of both mechanisms resulting from ricin-induced myocardial cell damage.

We believe this to be a direct effect of the ingested toxin, as we did not find any evidence of relevant ischaemia. There were no signs of ST-segment elevation at any time, and only mild elevation of cardiac biomarkers developed after the ventricular arrhythmia.

Re-entry, which has also been described as a possible pathomechanism of bidirectional VT, seems unlikely in our case due to the absence of structural heart disease facilitating macro- or micro-re-entry circuits.

Furthermore, the failure to terminate the arrhythmia with electrical cardioversion supports the hypothesis of sustained automaticity, possibly combined with triggered activity.

Bidirectional VT has been reported in the context of digoxin intoxication, where inhibition of the Na^+^/K^+^-ATPase leads to intracellular sodium accumulation, secondary calcium overload via the Na^+^/Ca^2+^ exchanger, and the development of delayed afterdepolarizations with triggered activity.^2,7^

Similarly, in catecholaminergic polymorphic ventricular tachycardia (CPVT), mutations in the ryanodine receptor (RyR2) and calsequestrin (CASQ2) gene predispose to abnormal calcium release under adrenergic stimulation, again resulting in delayed afterdepolarizations and triggered activity.^7^

These mechanisms demonstrate that dysregulated intracellular calcium handling, whether by toxin-induced Na^+^/K^+^-ATPase inhibition, inherited channel dysfunction, or direct myocardial cell damage, can produce the electrophysiological substrate for bidirectional VT.

In our case, we could not achieve rhythm control using specific measures recommended by current ESC guidelines, highlighting the unique pathophysiology of ricin-induced arrhythmia.^1^

There are some limitations to this case that should be acknowledged.

Ricin levels in the blood could not be quantified, which limits the ability to directly correlate toxin burden with the observed arrhythmia.

The specific intervention responsible for terminating the arrhythmia cannot be clearly identified, given the simultaneous use of supportive measures and antiarrhythmic therapy.

Finally, although unlikely based on the clinical course and follow-up, a genetic predisposition such as CPVT-related variants cannot be completely ruled out.

## Conclusion

This case highlights the potential for life-threatening arrhythmias following ricin intoxication. Clinicians should be aware of this possible complication and consider early supportive care and rhythm monitoring in affected patients. Although no targeted treatment was effective in our case, supportive management allowed for complete recovery.

Despite the limitations, the case provides valuable insight into a previously unreported arrhythmic manifestation of ricin intoxication.

## Lead author biography


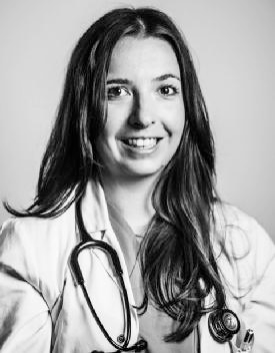


Dr. Anna SteinacherI=C2=B4m an attending cardiologist at the department of cardiology at Klinik Floridsdorf in Vienna.

## Data Availability

The data underlying this case report will be shared on reasonable request to the corresponding author.
